# Hair-based rapid analyses for multiple drugs in forensics and doping: application of dynamic multiple reaction monitoring with LC-MS/MS

**DOI:** 10.1186/s13065-014-0073-0

**Published:** 2014-12-13

**Authors:** Iltaf Shah, Andrea Petroczi, Martina Uvacsek, Márta Ránky, Declan P Naughton

**Affiliations:** School of Life Sciences, Kingston University, Kingston-upon-Thames, Surrey, UK; Faculty of Physical Education and Sports Sciences, Semmelweis University, Budapest, Hungary; Eötvös Lóránd University, Faculty of Education and Psychology, Budapest, Hungary

**Keywords:** Dynamic MRM, LC-MS/MS, Human hair, Controlled drugs, Doping, Forensics, Toxicology, Drugs of abuse

## Abstract

**Background:**

Considerable efforts are being extended to develop more effective methods to detect drugs in forensic science for applications such as preventing doping in sport. The aim of this study was to develop a sensitive and accurate method for analytes of forensic and toxicological nature in human hair at sub-pg levels.

**Results:**

The hair test covers a range of different classes of drugs and metabolites of forensic and toxicological nature including selected anabolic steroids, cocaine, amphetamines, cannabinoids, opiates, bronchodilators, phencyclidine and ketamine. For extraction purposes, the hair samples were decontaminated using dichloromethane, ground and treated with 1 M sodium hydroxide and neutralised with hydrochloric acid and phosphate buffer and the homogenate was later extracted with hexane using liquid-liquid extraction (LLE). Following extraction from hair samples, drug-screening employed liquid chromatography coupled to tandem mass spectrometric (LC-MS/MS) analysis using dynamic multiple reaction monitoring (DYN-MRM) method using proprietary software. The screening method (for > 200 drugs/metabolites) was calibrated with a tailored drug mixture and was validated for 20 selected drugs for this study. Using standard additions to hair sample extracts, validation was in line with FDA guidance. A Zorbax Eclipse plus C18 (2.1 mm internal diameter × 100 mm length × 1.8 μm particle size) column was used for analysis. Total instrument run time was 8 minutes with no noted matrix interferences. The LOD of compounds ranged between 0.05-0.5 pg/mg of hair. 233 human hair samples were screened using this new method and samples were confirmed positive for 20 different drugs, mainly steroids and drugs of abuse.

**Conclusions:**

This is the first report of the application of this proprietary system to investigate the presence of drugs in human hair samples. The method is selective, sensitive and robust for the screening and confirmation of multiple drugs in a single analysis and has potential as a very useful tool for the analysis of large array of controlled substances and drugs of abuse.

## Introduction

Hair testing is a convenient, tamper resistant and non-invasive technique for the analysis of many controlled drugs and drugs of abuse as compared to blood tests and urinalysis [1]. Hair testing can be used to screen for the parent drug and for metabolites and could be used to complement urinalysis [1-5]. In addition to urine tests, for the past three decades hair analysis has been employed to detect chronic social drug use and in the fight against doping in sport. Longer term histories of drug use can be detected as hair grows by approximately 1 cm per month [6-8]. Hair analysis provides a wider window of detection thus hair samples of a few centimetre lengths will provide more accurate information of drug use than blood and urine and it has good potential for out of competition doping [9-12]. Most controlled drugs and drugs of abuse are integrated into the hair matrix in a number of ways: (i) an endogenous-exogenous pathway; transfer or absorption of drug molecules into hair duct in the form of sweat and sebum from transdermal secretion, or (ii) by an endogenous pathway; the drug molecules diffuse into growing hair from circulatory system using passive transport [13-17]. Dosage, bioavailability, physiochemical properties and pharmacokinetics also affect the process of drug incorporation into the hair matrix. The concentration of drug detected in hair is also influenced by an individual’s metabolic pathway, cosmetic treatments and hair pigmentation. Hair also favours the incorporation of undissociated basic drugs due to carrying no net charge due to an isoelectric pH of about 6. Furthermore, due to the less polar nature of the parent drugs, its incorporation into the keratin matrix is favoured in contrast to their metabolites. While in urine it is the other way around and relatively low concentrations of parent drugs are excreted in urine as compared to their metabolites [18,19].

Doping in sport both in and out of competition is a persistent problem and therefore it is highly desirable to have a high throughput multi-analyte mass spectrometry method for the detection of controlled drugs and drugs of abuse in hair. Gas chromatography–mass spectrometry (GC-MS), requires additional derivatisation and lacks the required sensitivity to simultaneously detect large numbers of drugs in hair samples. Liquid chromatography- mass spectrometry (LC-MS/MS) when used in Multiple Reaction Monitoring (MRM) mode has been a powerful tool for detecting and confirming the presence of drugs in complex biological matrices. Recently, researchers have reported the hybrid triple-quadrupole mass spectrometer (QTrap) to screen and confirm 300, 100 and 88 drugs in human blood samples, respectively [20-26]. While others reported a 3200 Q Trap(R) LC-MS/MS system for analysis of 700 and 301 drugs in serum and urine samples [27,28]. A set of over 500 negative-ion MS–MS spectra was collected from three libraries applied in screening and systematic toxicological analysis is also reported [29]. Moreover, the mass spectra characteristics of more than 2,500 illegal drugs and metabolites have also been measured in urine and plasma using hybrid quadrupole time-of-flight mass spectrometry (LC-QTOF-MS) [30,31]. All of the above techniques are limited to human blood or urine samples. Furthermore, the LOD for most of these drugs were not suitable for doping control purpose as the methods of detections were not sensitive enough. However, there is an analytical method of metabolomic approach used for hair analysis using time of flight detector (TOF) and High resolution mass spectrometry (HRMS), which is limited to metabolite analysis and not the actual compounds and hence not widely used [32,33]. An UPLC-TOF-MS method for simultaneous screening and quantification of 52 drugs in hair was also developed and validated but the analysis of drugs were limited and only 15 autopsy hair samples were tested using this method [34].

The aim of this paper was (i) to develop a hair-based multi-drug/metabolite assay using LC-MS/MS with a dynamic multiple reaction monitoring (DYN-MRM) [35,36] method using a proprietary software, for both screening and validated confirmatory analyses, and (ii) to apply the method to hair samples for drugs and metabolites relevant to social drug use and doping in sport.

## Materials and methods

### Reagents and chemicals

LC/MS grade acetonitrile and deionised water together with formic acid, ammonium formate, dichloromethane, sodium hydroxide, hydrochloric acid, pentane, sodium hydrogen phosphate heptahydrate, and sodium phosphate monobasic dihydrate were obtained from Sigma-Aldrich (Poole-Dorset, UK). All drugs including nandrolone, stanozolol, testosterone, boldenone, cocaine, benzoylecgonine, cocaethylene, amphetamine, n-desmethyselegiline, ephedrine, methamphetamine, 3,4-methylenedioxy-N-methylamphetamine (MDMA), 3,4-methylenedioxyamphetamine (MDA), tetrahydrocannabinol (THC), 11-hydroxy-THC (11-OH-THC), THC-carboxylic acid-(THC-COOH), ketamine, norketamine, clenbuterol, propranolol, terbutaline, salbutamol, morphine, codeine and phencyclidine (PCP) together with internal standards stanozolol-d_3_, cocaine-d_3_, MDMA-d_5_, THC-d_3_, codeine-d_3_, ketamine-d_4_ and PCP-d_5_ were all purchased from LGC standards (Teddington, UK).

### Calibrant and stock solutions

Stock solutions of individual analytes and internal standards were prepared by dissolving or diluting it in methanol obtaining a concentration of 1 mg/mL. Individual stock solutions were diluted to prepare a standard mixture solution by mixing individual drug in methanol arriving at a concentration of 1 μg/mL. Moreover, working solutions of the individual standards were also prepared in methanol at various concentrations. The concentration of the working solution of internal standards mixture used was 10 ng/mL. Calibrants and quality control samples were prepared using blank hair. All standard stock solutions of forensic and toxicological interest were prepared in methanol at a concentration of 1 mg/mL. Furthermore, a bespoke working solution test mixture was prepared from dilution of stock solutions in-house at a concentration of 10 ng/mL. The test mix prepared, consisted of a representative range of forensic analyte classes of 27 components which is used to check the sensitivity and accuracy of the new dynamic MRM assay. All solutions were stored in amber plastic tubes at −80°C until further analysis.

### Sample preparation and analysis

Hair samples (50 mg) were collected from 233 volunteers as part of a research project investigating the discriminatory power of drug- and doping-related explicit and implicit social cognitive measurements in behavioural context indexed on the presence or absence of the target drugs in hair. On the average the hair samples were 3 cm in length (circa 120 mg in weight). The hair samples were cut directly from the vertex posterior of the head where it was possible. The hair samples were stored in labelled, sealed paper envelopes. The study was approved by the Kingston University Research Ethics Committee. Blank hair samples were obtained from healthy volunteers with no previous drugs use.

### Extraction of hair samples and screening using dynamic MRM-LC-MS/MS mode

The Agilent Technologies forensic and toxicology dynamic MRM-LC-MS/MS kit was used to screen hair samples for all controlled substances and drugs of abuse of interest by employing a single point calibration curve. For extraction, the hair samples were decontaminated using dichloromethane then milled in a mini ball mill (Fritsch and Gerhardt UK Ltd); 20 mg hair was weighed out and incubated with 1 M sodium hydroxide at 95°C for 5 minutes. The mixture was neutralised with 1 M hydrochloric acid followed by addition of 2 mL of 0.2 M phosphate buffer pH 7 [6]. The sample was further extracted using hexane and 2 μL injected on to the LC-MS/MS system. The kit was found to be a good and convenient starting point for the screening of a large array of toxicological and forensic analytes. As compared to the multiple reactions monitoring (MRM), dynamic MRM has an associated delta retention time window (Del RT) where it dynamically turns on and off without affecting the total cycle time. But some time compromises between, cycle time, dwell time and the total number of MRM transitions are required in a large number of analytes screening assays. For screening purposes we have used mostly 4 MRM transitions for each compound.

### Confirmation of extracted hair samples

For quantitative confirmatory analysis of positive hair samples a 6–8 point calibration curve was used along with quality control samples. A further 2 μL of extracted hair sample was injected for quantitative analysis using DYN-MRM-LC-MS/MS. Only the two most abundant MRM transitions were used for confirmatory analysis. Of the two, the most sensitive transition was used as quantifier ions and the other transition as qualifier ions, the ratios of which are indicative of the analyte of interest.

### Instrumentation

The LC–MS/MS system comprised of a 1260 infinity LC system (Agilent Technologies UK) coupled to a 6430 triple quadrupole mass spectrometer (Agilent Technologies UK). The LC system consisted of a 1290 infinity thermo-stated autosampler, degasser, binary pump and column heater. Agilent Masshunter Forensics and Toxicology dynamic MRM software kit was used in combination with Masshunter workstation software. An electrospray ionisation (ESI) source was employed for samples ionisation. The chromatographic separation was achieved using a Zorbax Eclipse plus C18 (2.1 mm × 100 mm, 1.8 μm) column. Column was heated to 65°C for good reproducibility. The analytical column was connected in tandem with a 0.2 μm inline filter to prevent it from blocking. Mobile phase consisted of a Solvent A [10 mM ammonium formate/0.02 M formic acid in water] and solvent B [0.02 M formic acid in acetonitrile].

The flow rate through the column was set at 0.4 mL/min. The gradient flow composition is described as follows. The mobile phase linear gradient was run with 90% solvent A for 0.5 minutes and 10% solvent B, decreasing to 50% solvent A in 3 minutes, decreasing further to 5% solvent A and 95% solvent B, and held from 4 to 6 minutes and then returned to 90% of solvent A until 7 minutes, and stabilised until 8 minutes before next injection. The bypass configurations were set up for the mixer and damper when using this kit with our Agilent HPLC 1260 binary pump. This was to convert the pump to low delay volume mode to achieve better chromatography. The mass spectrometer was operated in positive ionization polarity at a spray capillary voltage of 3000 V. Sheath gas temperature and fow was 350°C, 12 L/min, nozzle voltage was 450 V, drying gas temperature and flow 300°C, 8 L/min, nebulizer gas pressure was 25 psi and fragmentor voltage was set to 150 V, Delta retention time was 1 for all ions in dynamic MRM mode. The total run time was 8 min together with 2 min post time equilibration. The acquired dynamic MRM transitions, fragmentor voltages and collision energies of quantified analyte are given in Table 1 [36].

**Table 1:** **Chromatogarphic and spectrometric characteristics of analytes selected for validation for confirmatory analyses**

**Compound name**	**Precursor ion**	**Product ion**	**Fragmentor**	**Collision energy**
Nandrolone	275.1	257	105	5
		109.1		21
Stanozolol	329.1	175.2	105	10
		107.1		34
Boldenone	287.2	135.1	105	5
		121.1		20
Cocaine	304.2	182.1	138	17
		77		61
Benzoylecgonine	290.1	168.1	118	17
		77		60
Amphetamine	136.1	119.1	66	5
		91		17
Methamphetamine	150.1	91	92	17
		65.1		45
Ephedrine	166.1	148.1	81	9
		115		25
MDMA	194.1	163	97	9
		105		25
THC	315.2	123.3	150	30
		193.2		20
THC-COOH	345.2	299.2	140	18
		327.2		18
11-Hydroxy THC	331.2	313.2	112	9
		193.1		21
Morphine	286.2	165.1	158	41
		152		60
Codeine	300.2	165.1	158	45
		58.1		29
Clenbuterol	277	203.1	100	15
	240	166	90	15
Propranolol	260	115.9	110	22
Salbutamol	240	73.9	90	22
Terbutaline	226.2	125.1	100	20
		152.1		20
PCP	244.2	91	86	41
		86.1		9
Ketamine	238.1	220.1	105	11
		125		11

### Method validation

Given the necessity to adopt tailored test mixtures based on the drug profile of interest it was necessary to validate the methods for confirmation of positive drugs. Repeated injections (n = 6) were performed on a single day to establish the intra-day precision (% CV), while the inter-day precision was evaluated on several days. The accuracy was expressed as the ratio of the compound added to the measured (mean value/nominal value) × 100. Extraction efficiency of the compounds was determined by spiking blank hair samples with 10 pg/mg drugs. Absolute recoveries were determined by comparing the concentrations obtained from peak height ratios of extracted hair samples with added concentrations (standard addition method was used for the analysis of testosterone). Relative recoveries were determined by comparing the % amount of drug recovered from hair matrix against detector response for extracted standards, utilising three different concentrations of quality controls. Stability checks were performed by carrying out three freeze/thaw cycles (between room temperature to −20 ± 5°C), in hair samples by spiking blank hair with three different concentrations of quality controls (10, 25 and 50 pg/mg).

## Results and discussion

The MassHunter Forensics and Toxicology Dynamic MRM Database Kit was able to screen for 300 analytes of Forensics and Toxicology nature with enhanced sensitivity, all in a single LC/MS analytical run. The Kit also helped to minimize method development for this analysis. The kit came equipped with, MRM transitions for analytes of forensics and toxicological nature along with their optimized fragmentor voltages and collision energy settings. During method development, these MRM transitions were imported from the database to the MassHunter Data Acquisition program where these transitions were used for screening and confirmation purpose. These MRM transitions were also compared against MRM transitions for infused analytes. The Dynamic MRM feature of Agilent Triple Quadrupole mass spectrometer provided us with an adaptive MRM data collection methodology where the instrument was able to collect data only during a predetermined time window. The advantage is that more than one compound/MRMs can be analyzed in a single run through the Dynamic MRM feature, without losing data quality. The Agilent MassHunter software was used for data generation. Table 1 shows the precursor and product ions of the confirmed analytes along with their fragmentor voltages and collision energies. The protonated molecules [M + H]^+^ was identified as the precursor ions and the diagnostic product ions were monitored in dynamic multiple reactions monitoring (MRM) mode.

**Figure 1:**
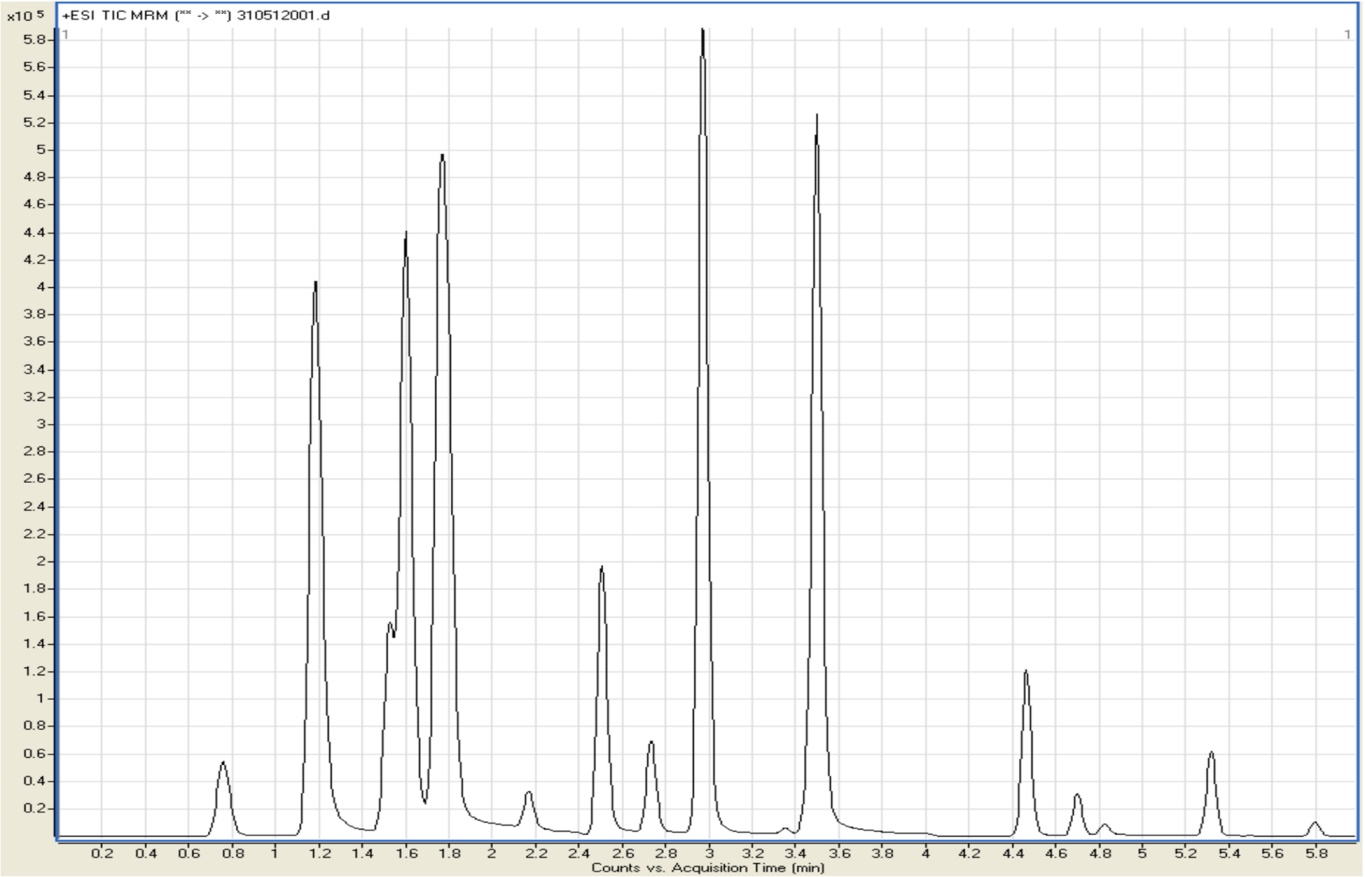
**Chromatogram showing peaks for 27 spiked drugs in a single extract of human hair using DYN-MRM-LC-MS/MS.** (See text for peak elution times).

Dynamic MS/MS transitions for the LC/MS Forensics and Toxicology test mixture and their chromatographic evaluation could not be achieved because of the unavailability of this kit in the UK, and due to Home office licensing restrictions in the UK. The commercially available standard Dyn-MRM kit mixture was comprised of about 24 drugs (codeine, oxycodone, amphetamine, methamphetamine, MDA, MDMA, MDEA, hydrocodone, strychnine, cocaine, heroin, meperidine, trazodone, PCP, oxazepam, nitrazepam, verapamil, methadone, lorazepam, alprazolam, temazepam, proadifen, diazepam and THC). Because of its unavailability in UK, we had to tailor make a test mixture which consisted of the following 21 drugs of interest along with 6 internal standards (nandrolone, stanozolol, boldenone, testosterone, cocaine, benzoylecgonine, amphetamine, methamphetamine, ephedrine, MDMA, THC, THC-COOH, 11-hydroxy THC , morphine, codeine, clenbuterol, salbutamol, propanolol, terbutaline, PCP, ketamine and internal standards like stanozolol-d_3_, amphetamine-d5, methamphetamine-d5, MDMA-d5,THC-d_3_ and cocaine-d_3_). The chromatogram in Figure 1 shows the standard mixture of 27 drugs spiked in human hair extracted and analysed using DYN-MRM-LC-MS/MS method.

The retention times of 21 spiked drugs along with their 6 internal standards in human hair, along with 6 internal standards spiked in human hair where delta retention time was 1, are as follows: Peaks = Morphine (0.71 min), terbutaline (0.75), amphetamine-d_5_ (1.67), amphetamine (1.67), methamphetamine (1.76), methamphetamine-d_5_ (1.76), MDMA (1.92), 5-MDMA (1.92), ketamine (2.3), benzoylecgonine (2.67), clenbuterol (2.73), cocaine (2.98), cocaine-d_3_ (2.98), boldenone (3.22), THC (3.35), THC-d_3_ (3.35), nandrolone (3.4), PCP (3.5), codeine (3.54), ephedrine (3.71), salbutamol (3.95), propranolol (4.32), testosterone (4.7), stanozolol (4.81), stanazolol-d_3_ (4.81),THC acid (5.29), 11-hydroxy THC (5.3). The Delta retention time for all analytes was 1.

**Figure 2:**
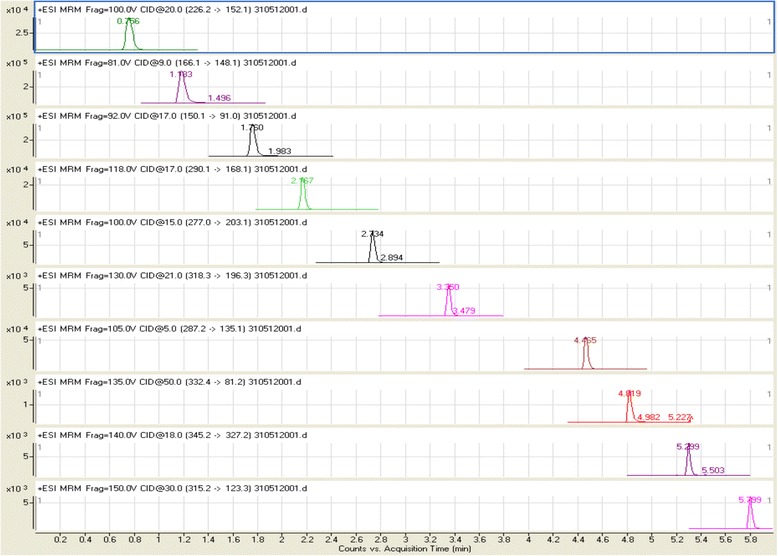
**DYN-MRM-LC-MS/MS chromatogram showing delta retention time (Del RT,Δt) window for 10 spiked drugs of abuse in a single extract of human hair sample.** From top: terbutaline, ephedrine, methamphetamine, benzoylecgonine, clenbuterol, THC-d_3_, boldenone, stanozolol-d_3_, THC acid and cannabidiol (THC).

The following representative chromatogram (Figure 2) shows the importance of the short retention time windows functionality (Del retention time, Δt) for the entire run time. In this example, ten drugs were spiked into a hair sample, extracted and analysed using DYN-MRM-LC-MS/MS method.

### Validation results

Intra-day and inter-day precision and accuracy are shown in Table 2. Drug free blank hair was analysed to evaluate the specificity of analytical method. Endogenous drugs (e.g. testosterone) solutions were prepared in human hair samples using the standard additions method. The absolute recoveries of the analytes and internal standards were ranging from 98.2 to 98.7% and 94.3 to 99.6%, respectively. Relative recoveries for analytes and internal standards ranged from 99.8 to 102.2% and 99.8 to 99.9% respectively. In the Table, r^2^ represents the linear regression; all the units are represented in pg/mg units. The assay showed excellent sensitivity (LOD = 0.1 pg) and linearity. Where limit of quantitation ranged between 0.1-1 pg/mg. Calibration linearity was not less than (R^2^ = 0.9987) for the spiked analytes.

**Table 2:** **Summary of assay validation results**

**Drugs in (pg/mg)**	**Conctrn. QC's**	**Linear range**	**LOD**	**r** ^**2**^	**Inra-day (n = 6)**		**Inter-day(n = 6)**	
					**Precision, % CV**	**Accuracy,%**	**Precision, % CV**	**Accuracy,%**
Nandrolone	10	0.1-100	0.05	0.9998	3.23	100.1	4.82	99.8
	25				1.96	100.4	1.95	99.4
	50				1.41	99.9	1.39	100.2
Stanozolol	10	0.1-100	0.05	0.9995	6.3	102.1	9.4	101.2
	25				10.2	98.5	7.1	99.8
	50				8.6	99.4	3.2	101.3
Boldenone	10	0.5-100	0.25	0.9998	4.9	99.9	3.4	101.1
	25				3.3	100.3	2.7	99.8
	50				2.5	99.7	2.3	98.7
Cocaine	10	0.5-100	0.25	0.9998	3.5	98.9	3.2	99.8
	25				4.3	99.4	3.3	98.9
	50				2.9	99.9	2.3	97.8
Benzoylecgonine	10	0.5-100	0.25	0.9987	3.5	107.2	4.7	100.5
	25				2.1	102.3	10.9	102.6
	50				3.3	100.1	8.9	99.9
Amphetamine	10	0.25-100	0.1	0.9979	7.1	98.8	3.2	100.5
	25				5.9	99.6	2.7	100.1
	50				3.2	89.9	3.2	100.3
Methamphetamine	10	0.5-100	0.25	0.9988	5.2	98.3	5.3	100.2
	25				2.3	102.6	2.1	103.3
	50				4.1	100.9	3.6	108.1
Ephedrine	10	1-100	0.5	0.9945	3.8	99.3	4.3	101.2
	25				3.5	98.7	2.1	99.8
	50				3.2	101.2	3.2	99.9
MDMA	10	0.5-100	0.25	0.9968	5.1	99.4	5.1	100.3
	25				2.4	103.2	3.3	110.2
	50				3.7	101.6	6.2	104.2
THC	10	1-100	0.5	0.9996	2.1	107.3	4.3	109.2
	25				3.2	110.2	2.6	105.3
	50				5.1	101.3	3.9	103.2
THC-COOH	10	1-100	0.5	0.9992	4.3	100.1	3.3	101.1
	25				3.6	98.9	3.9	99.9
	50				2.9	98.6	4.3	98.9
11-OH-THC	10	0.5-100	0.25	0.9998	3.6	89.8	5.3	90.5
	25				2.1	89.9	5.9	89.6
	50				5.3	90.9	2.1	99.8
Morphine	10	0.5-100	0.25	0.999	4.2	89.3	3.2	90.9
	25				6.3	88.9	5.3	91.8
	50				3.9	100.9	2.1	99.5
Codeine	10	0.5-100	0.25	0.999	2.9	98.9	1.9	99.5
	25				8.3	99.5	5.6	99.3
	50				2.4	99.3	1.5	9.8
Clenbuterol	10	0.5-100	0.25	0.9989	5.1	114.2	4.6	104.3
	25				2.1	89.9	1.3	103.4
	50				1.2	99.9	1.3	105.6
Salbutamol	10	1-100	0.5	0.9998	2.1	99.9	1.9	102.3
	25				1.9	98.4	1.8	101.2
	50				3.2	100.5	2.1	101.1
Propranolol	10	0.1-100	0.05	0.9999	2.2	99.7	1.9	100.5
	25				2.6	89.9	2.5	99.78
	50				1.1	98.9	1.5	98.5
Terbutaline	10	0.5-100	0.25	0.9997	1.7	100.8	1.9	99.9
	25				1.2	99.9	2.1	100.5
	50				2.2	100.5	1.9	102.3
PCP	10	0.5-100	0.25	0.9998	1.4	102.3	2	100.3
	25				2.1	101.3	3.5	100.1
	50				3.2	105.1	1.5	100.5
Ketamine	10	0.5-100	0.25	0.9996	1.6	104.2	1.1	98.9
	25				2.6	102.3	2.1	99.9
	50				2.4	104.2	2.2	99.6

### Application in hair-analysis

**Figure 3:**
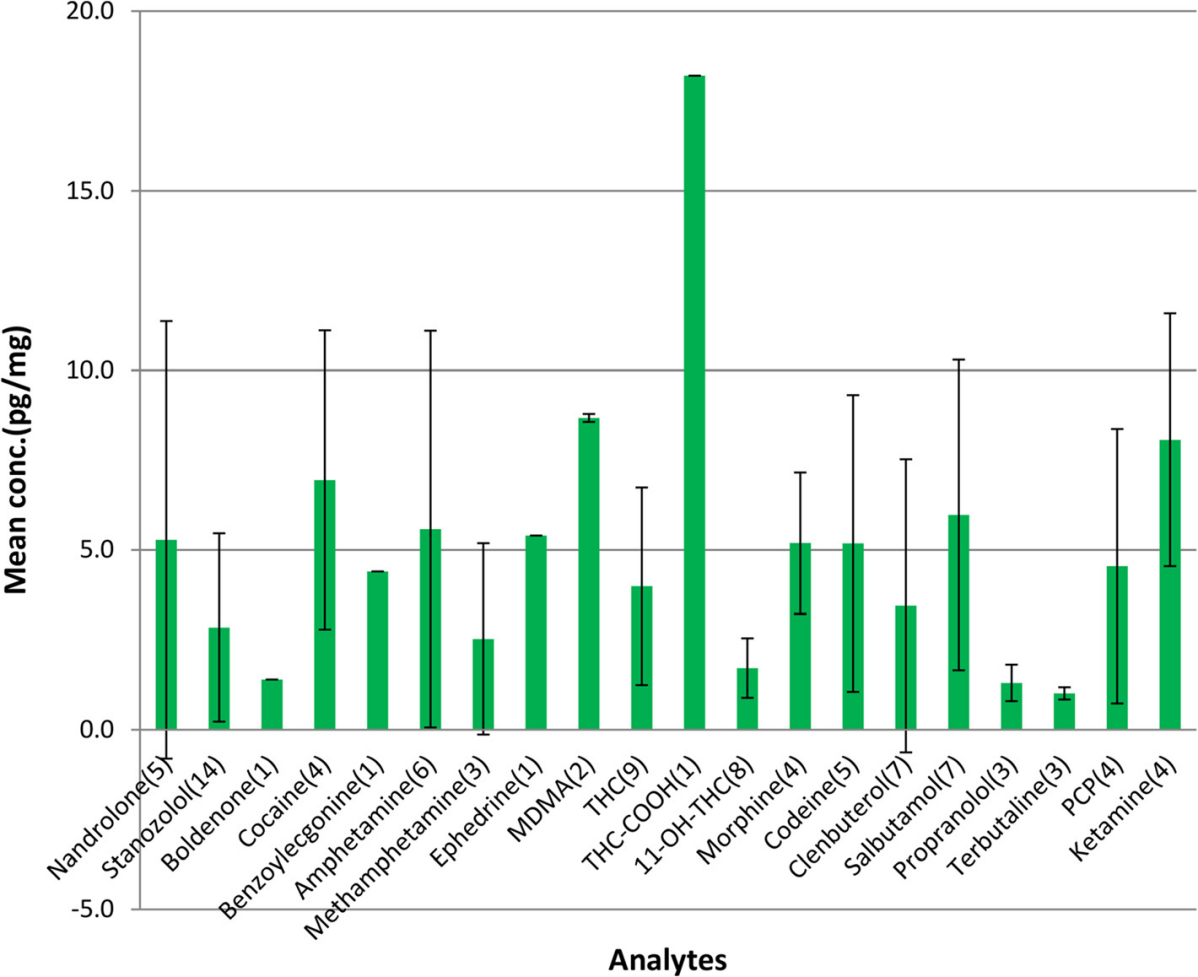
**Mean concentrations of analytes along with the frequency of positive occurrences (shown in brackets).** The error bars represents standard deviation.

For this study the analysis results of 233 hair samples showed that about 70 volunteers were confirmed positive for some of 20 different of drugs of forensic nature, mainly steroids and drugs of abuse. Out of these 70 only one volunteer was positive for 5 drugs, 2 volunteers were positive for four different drugs and 11 volunteers were positive for 2 drugs at the same time. The rest of the 56 volunteers were positive for 1 drug each. The range of drugs found in hair and their frequencies are given in Table 3 and Figure 3.

**Table 3:** **The quantitative range and frequency of occurrence of confirmed drugs in human hair**

**Drugs**	**Frequency of occurrences**	**Range of drugs found in hair (pg/mg)**
Nandrolone	5	0.12 − 15.28
Stanozolol	14	0.36 − 8.24
Boldenone	1	1.4
Testosterone	233	1.19 − 4.1
Naltrexone	2	8.6 − 16.6
Cocaine	4	1.2 − 10.3
Benzoylecgonine	1	4.4
Amphetamine	6	0.28 − 13.6
Methamphetamine	3	0.884 − 5.6
Ephedrine	1	5.4
MDMA	2	8.6 − 8.76
THC	9	1.2 − 8.4
THC-COOH	1	18.2
11-OH-THC	8	0.61 − 3.1
Morphine	4	1.9 − 10.8
Codeine	5	3.4 − 8.3
Clenbuterol	7	0.95 − 9.2
Salbutamol	7	1 − 12.52
Propranolol	3	1.12 − 12.4
Terbutaline	3	0.79 − 1.8
PCP	4	0.89 − 1.13
Ketamine	4	1.3 − 9.6

### Benefits of dynamic-MRM over MRM technique

Multiple reactions monitoring (MRM) mode has been widely used as a suitable way of conducting quantitative analysis using triple quadrupole mass spectrometry, but the limitation of this method is the use of a series of time segments to monitor MRM transitions. In order to analyse very low quantities of hundreds of compounds of forensic and toxicological nature, in a wide variety of matrices, dynamic MRM is useful. Dynamic MRM method development and modifications are a lot easier and more reliable and provide better quality data compared to traditional time segments in MRM mode. A further benefit of this method is that, rather than performing scans for all the analytes during the entire method, the mass spectrometer only monitors MRM transitions for the analytes that elute in that particular time segment. The result is that there are fewer transitions recorded during each MS scan, allowing the mass spectrometer to use a longer dwell time and more data points per peak. In other words, analytes are only monitored while they are eluting from the LC to focus detection. This approach also allows total exclusion of background and interferences problems. These qualities made it possible to use dynamic MRM mode to screen for about 20 drugs of forensic and toxicological nature in hair samples just over 8 minute time period. Since a quantifier ion and a qualifier ion were necessary for confirmatory purposes, a total of about 40 dynamic MRM transitions were employed during the analysis, covering classes of analytes such as steroids, amphetamines, cannabinoids, cocaine’s and bronchodilators etc.

### Advantages of hair-dynamic MRM drug analyses method

This study reports the application of a hair-based multi-drug analytical approach which has high sensitivity and accuracy and is capable of analysisng some 200 drugs/metabolites in one chromatographic run. The benefits of the novel method over current approaches include: i) long-term such as quarterly detection windows (for 3 cm hair samples) and ease of ‘non-invasive hair’ collection, reduced infection risk and storage demands, ii) using Dyn-MRM software affords the ability to screen for some 200 compounds of interest simultaneously in one chromatographic run (i.e. 8 min) using a test mixture to calibrate the LC component, iii) develop a full validation for compounds of interest and thus perform full confirmation analysis, iv) adapt the software to add new compounds of interest.

It should be noted that the focus of this study was on the range of drugs and their metabolites listed. For this reason the calibrant test mixture was prepared and validation steps were completed with these selected compounds. The method also allows screening for any of the other 200 plus drugs/metabolites from spectral library and full validation can be added using reference and standard material.

## Conclusion

This study reports the development and validation of a method to analyse drugs of forensic and toxicological nature in human hair samples. The method is selective, sensitive and robust for the screening and confirmation of multiple drugs in a single analysis and will be very useful tool for the analysis of large array of controlled substances and drugs of abuse. This is the first report of the application of this proprietary system to drugs of abuse in hair samples. In this example, it employs the Dyn-MRM software to screen and confirm for 20 selected analytes (via full validation) of forensic interest present in the hair samples in a single chromatographic run.
